# Molecular Phylogeny and Morphological Analysis Support a New Species and New Synonymy in Iranian *Astragalus* (Leguminosae)

**DOI:** 10.1371/journal.pone.0149726

**Published:** 2016-03-09

**Authors:** Ali Bagheri, Ali Asghar Maassoumi, Mohammad Reza Rahiminejad, Frank R. Blattner

**Affiliations:** 1 Department of Biology, Faculty of Sciences, University of Isfahan, Isfahan, Iran; 2 Department of Botany, Research Institute of Forests and Rangelands, Tehran, Iran; 3 Leibniz Institute of Plant Genetics and Crop Research (IPK), Gatersleben, Germany; 4 German Centre of Integrative Biodiversity Research (iDiv) Halle-Jena-Leipzig, Leipzig, Germany; Field Museum of Natural History, UNITED STATES

## Abstract

As a result of a taxonomic and phylogenetic revision of *Astragalus* section *Hymenostegis* we identified a new species of *Astragalus* from northwestern Iran, namely *A*. *remotispicatus* spec. nov., which is described and illustrated here. It is morphologically similar to *A*. *karl-heinzii* in possessing a lax inflorescence. Phylogenetic inference of the nuclear ribosomal DNA internal transcribed spacer (ITS) region support *A*. *remotispicatus* as a clearly distinct species within the lax-inflorescence group of this section. Also the placement of *A*. *sciureus* var. *subsessilis* was found to be wrong and this taxon should be treated as a synonym within *A*. *kohrudicus*.

## Introduction

*Astragalus* L. is with 2950 species the largest genus of flowering plants [1]. Section *Hymenostegis* Bunge is one of the large-sized and spiny sections of the genus in Iran with 59 recognized species, Iran, especially the northwest of the country, with about 75% of endemics is the center of diversity of this section [2]. Morphologically there can be two main groups discerned within this section: 1) the lax-inflorescence group and 2) the dense-inflorescence group [1,3,4,5,6]. In the first group the axis of the inflorescence is at least partly visible while the inflorescence axis of the second group is concealed by flowers. Five species within the lax-inflorescence group are discerned. One of them is *A*. *karl*-*heinzii* Maassoumi, which was described from Masuleh, northern Iran [7]. As a result of our recent fieldwork in the Zanjan province in northwestern Iran, some new populations of lax-inflorescence plants were discovered. Further examination showed that these specimens morphologically resemble *A*. *karl-heinzii*. The comparison of these individuals with the holotype of *A*. *karl-heinzii* showed however that the newly found plants are different from this species. This difference is also supported by sequences of the nuclear ribosomal DNA ITS region, including the spacers ITS1 and ITS2 together with the 5.8S rRNA gene.

**Fig 1:**
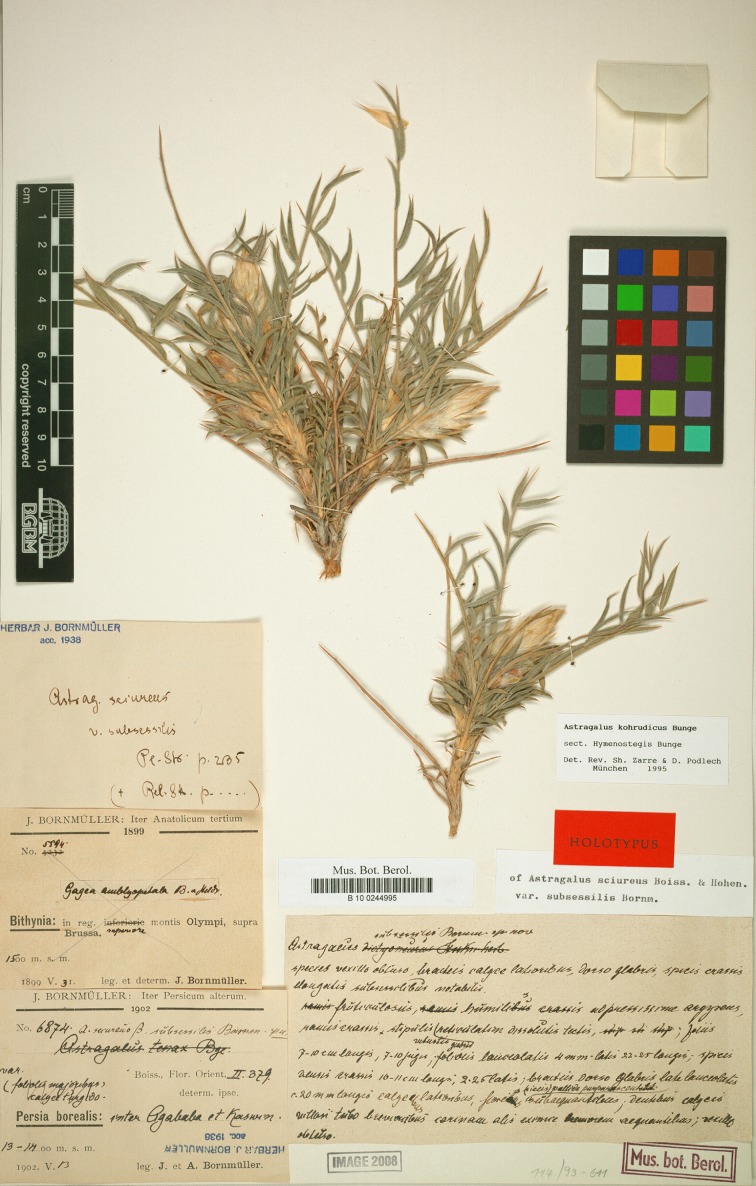
Holotype of *Astragalus sciureus* var. *subsessilis* = *A*. *kohrudicus* (digital barcode in B herbarium: B 10 0244995).

**Fig 2:**
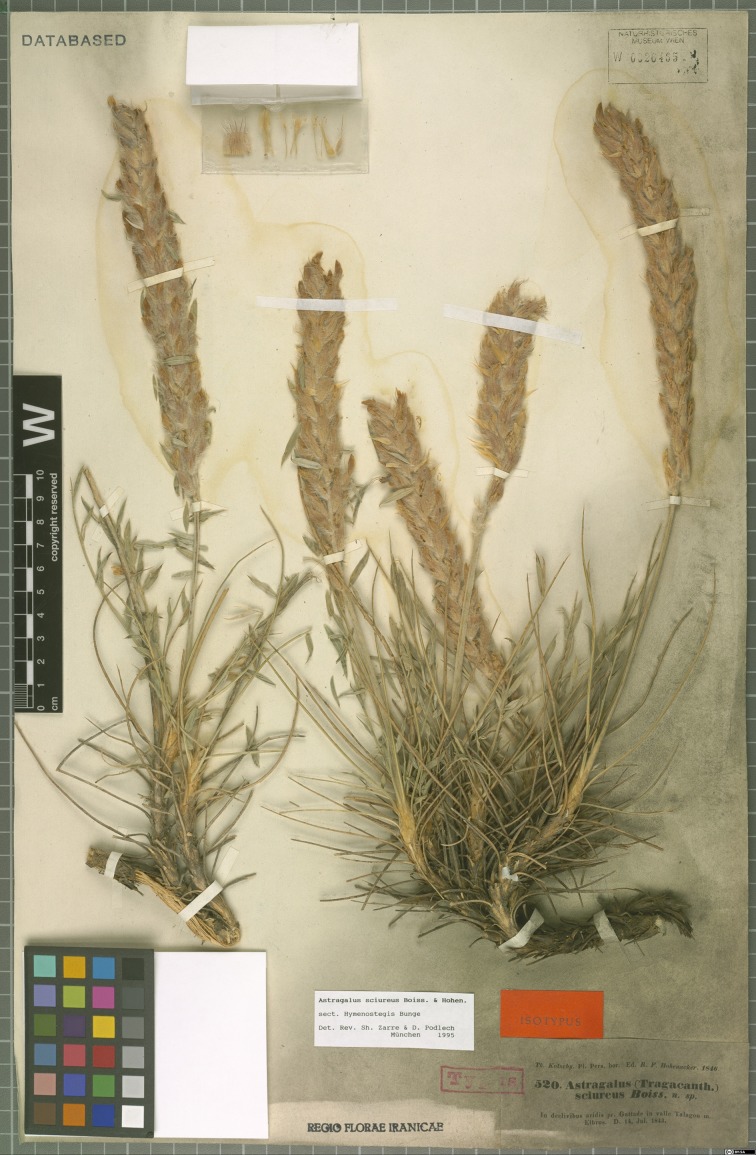
Isotype of *Astragalus sciureus* (digital barcode in W herbarium: W 0026465).

**Fig 3:**
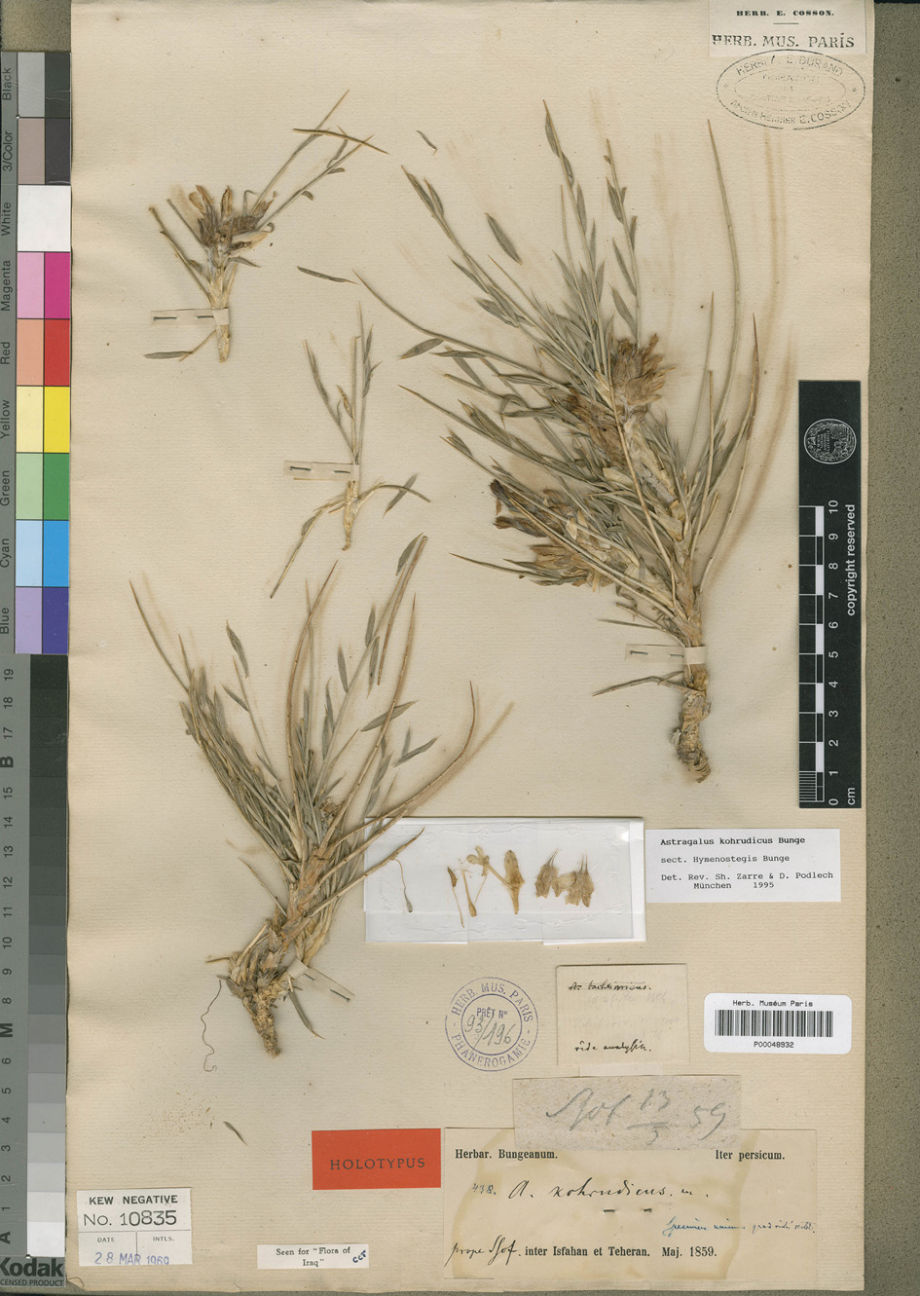
Holotype of *Astragalus kohrudicus* (digital barcode in P herbarium: P00048932).

*Astragalus sciureus* Boiss. & Hohen. is another member of the lax-inflorescence group of sect. *Hymenostegis*. In an account on *Astragalus* taxa collected from Alborz Mountains, Bornmüller [8] published the new variety “*subsessilis*” of *Astragalus sciureus*. Parsa [9] accepted Bornmüller’s decision. However, Rechinger et al. [10] in their fully revised treatment of this section and Maassoumi [4] in his checklist for *Astragalus* in the Old World disregarded this variety. The first author of this article noted some interesting points about the holotype of *A*. *sciureus* var. *subsessilis* (specimen number 6894, B). This specimen fitted well with the diagnosis of *A*. *kohrudicus* Bunge, as previously determined on the same sheet by two other taxonomists (Fig 1). Nevertheless, in a new approach toward the genus, Maassoumi [3] and Zarre & Podlech [11] included var. *subsessilis* in their revisions of sect. *Hymenostegis* as a synonym of *Astragalus sciureus*. This treatment was accepted by the following accounts on *Astragalus* including Podlech & Maassoumi [5], Podlech [12], and Podlech & Zarre [1]. As a result of a recent visit at the herbarium B and a study of the holotype of this variety and comparisons with the type specimens of *A*. *sciureus* (Fig 2) and *A*. *kohrudicus* (Fig 3) in the herbaria of B, W, MSB and P we decided to re-revise the taxonomic position of *A*. *sciureus* var. *subsessilis* using morphological and molecular data.

## Material and Methods

### Nomenclature

The electronic version of this article in Portable Document Format (PDF) in a work with an ISSN or ISBN will represent a published work according to the International Code of Nomenclature for algae, fungi, and plants, and hence the new names contained in the electronic publication of a PLoS article are effectively published under that Code from the electronic edition alone, so there is no longer any need to provide printed copies.

In addition, new names contained in this work have been submitted to IPNI, from where they will be made available to the Global Names Index. The IPNI LSIDs can be resolved and the associated information viewed through any standard web browser by appending the LSID contained in this publication to the prefix http://ipni.org/. The online version of this work is archived and available from the following digital repositories: (PubMed Central, LOCKSS etc).

### Specimens examined for the new *Astragalus* species

The morphological study was based on field collections and herbarium sheets (including type specimens) deposited in the herbaria TARI, HUI, MSB, M, B, and W. Morphological data of the unusual *Astragalus* populations from the Zanjan province in northwestern Iran (described here as a new species) were obtained from direct examination of the specimens. We provide a detailed morphological analysis between this species and *A*. *karl-heinzii*, because the new species exhibits morphological characters that mostly resemble this species. The examined taxa are listed in Table 1.

**Table 1:** Information of examined taxa in this study.

Taxon	Locality	Herb. No. & Coll.	Herbarium & Type status
***A*. *sciureus* var. *subsessilis***	Saudsch-Bulag inter Agababa et Kaswin, 1300–1400 m.	Bornmüller, 6874	B, Holotype
***A*. *sciureus***	Ad Gattade vallis Talagon montis Elbrus, without elevation	Kotschy, 520	W, MSB, Isotype
***A*. *sciureus***	Ghazvin, Alamut, Moallem Kelayeh, 1800 m.	Bagheri, 97946	TARI
***A*. *sciureus***	Karaj, Kondar, 2000 m.	Amin & Barazgan, 19326	W
***A*. *kohrudicus***	Sorkhe Hesar, Haraz road, 1400 m.	Amin & Bazargan 19018	W
***A*. *kohrudicus***	Tehran, Takestan, 1450 m.	Assadi & Mozzafarian, 36618	TARI
***A*. *kohrudicus***	Ghazvin to Zanjan, without elevation	Terme, 49783	W
***A*. *karl-heinzii***	Ardabil, Masuleh to Khalkhal, 2100 m.	Assadi, 86477	TARI, Holotype
***A*. *karl-heinzii***	Zanjan, Gheydar, Gheydar Mt., 2300 m.	Bagheri, 98274	TARI
***A*. *karl-heinzii***	Zanjan, Gheydar, Gheydar Mt., 2250 m.	Bagheri, 6617	TARI
***A*. *karl-heinzii***	Zanjan, Gheydar, Gheydar Mt., 2200 m.	Bagheri, 9	TARI
***A*. *remotispicatus***	Zanjan, Gheydar, Zarand, Zarand Mt., 2300 m.	Bagheri, 97932a	TARI, Holotype
***A*. *remotispicatus***	Zanjan, Gheydar, Zarand, Zarand Mt., 2300 m.	Bagheri, 97932b	TARI

### Specimens examined for *Asragalus sciureus* var. *subsessilis*

During his visit at the herbarium B, the first author of this article noted a close resemblance of the holotype of *A*. *sciureus* var. *subsessilis* to *A*. *kohrudicus*. In order to clarify the position of this variety herbarium sheets and field collections of *A*. *sciureus* and *A*. *kohrudicus* (including type specimens) were morphologically examined (Table 1).

### Molecular analysis

For the studied taxa as well as for *A*. *vaginans* DC., the sister taxon of the section *Hymenostegis*, and *Oxytropis rechingeri* Vassilcz. and *O*. *aucheri* Boiss. as outgroups, DNA was isolated from leaf tissue using a DNeasy Plant Mini Kit (Qiagen) according to the manufacturer’s instructions. The ITS region, including the spacers ITS1 and ITS2 together with the 5.8S rRNA gene, was amplified by polymerase chain reaction (PCR) using the primers ITS-A and ITS-B [13]. After purification of the amplicons on a Nucleofast Spin Plate (Macherey-Nagel) they were Sanger sequenced with an ABI 3730XL DNA sequencer (Applied Biosystems) using the amplification primers. Forward and reverse sequences of each individual were checked, manually edited if necessary, and combined into consensus sequences. The sequences were manually aligned. Sequence evolution models were evaluated in Paup* 4.0a146 [14] using the Bayesian Information Criterion (BIC) resulting in the Jukes-Cantor model of sequence evolution as best-fitting model. Bayesian phylogenetic analyses were conducted with this model in MrBayes 3.1.2 [15] running two analyses with four chains each for one million generations, sampling a tree every 500 generations. Evaluation of the analyses showed that both analyses had converged and arrived at similar likelihood values. The first 25% of sampled trees were discarded as burn-in. The remaining trees were summarized with MrBayes. In addition a parsimony analysis was conducted in Paup* using the heuristic search algorithm with TBR branch swapping. Support of clades was estimated by 500 bootstrap re-samples. A list of voucher specimens and GenBank accession numbers of the ITS sequences are given in Table 2.

**Table 2:** Information of examined taxa in molecular study.

Taxon	DNA source	GenBank accession No.
***A*. *sciureus* var. *subsessilis***	6874, B	KT894779
***A*. *sciureus***	520, MSB	KT894782
***A*. *sciureus***	97946, TARI	KT894781
***A*. *sciureus***	19326, W	KT894780
***A*. *kohrudicus***	19018, W	KT894776
***A*. *kohrudicus***	36618, TARI	KT894777
***A*. *kohrudicus***	49783, W	KT894778
***A*. *karl-heinzii***	86477, TARI	KT997422
***A*. *karl-heinzii***	98274, TARI	KT997423
***A*. *karl-heinzii***	6617, TARI	KT997424
***A*. *karl-heinzii***	9, TARI	KT997425
***A*. *karl-heinzii***	98260, TARI	KT997426
***A*. *remotispicatus***	97932a, TARI	KT997427
***A*. *remotispicatus***	97932b, TARI	KT997428
***A*. *vaginans***	2440, GAZI	AB908466.1
***O*. *rechingeri***	51253, TARI	AB741305.1
***O*. *aucheri***	55104, TARI	AB051908.1

## Results

### Morphological analysis

Based on the morphological analysis we here describe the recently detected lax-inflorescence plants from Zanjan as a new species of the genus *Astragalus* sect. *Hymenostegis*. These analyses also showed that *Astragalus kohrudicus* differs from *A*. *sciureus* by several diagnostic morphological features (Table 3), which make them easily distinguishable from each other. *Astragalus sciureus* var. *subsessilis* clearly falls within the character space of *A*. *kohrudicus* and should be treated as a synonym of this taxon.

**Table 3:** Differences between *Astragalus kohrudicus* (including *A*. *sciureus* var. *subsessilis*) compared to *A*. *sciureus*.

Species / Characteristic	*A*. *kohrudicus*	*A*. *sciureus*
Stipules texture	chartaceous	hyaline-membranous
Stipules length	9–15 mm long	15–27 mm long
Peduncles length	0.5–1.5 cm long	4–20 cm long
Peduncles hairs	erect	appressed to subappressed
Inflorescence shape	ovoid to cylindrical	cylindrical
Inflorescence length	5–15 cm long	13–25 cm long
Inflorescence density	dense (densely many-flowered)	lax (loosely many-flowered)
Wings	19–28 mm long	15–23 mm long
Keel	19–26 mm long	14–20 mm long

### Molecular studies of ITS sequences

**Fig 4:**
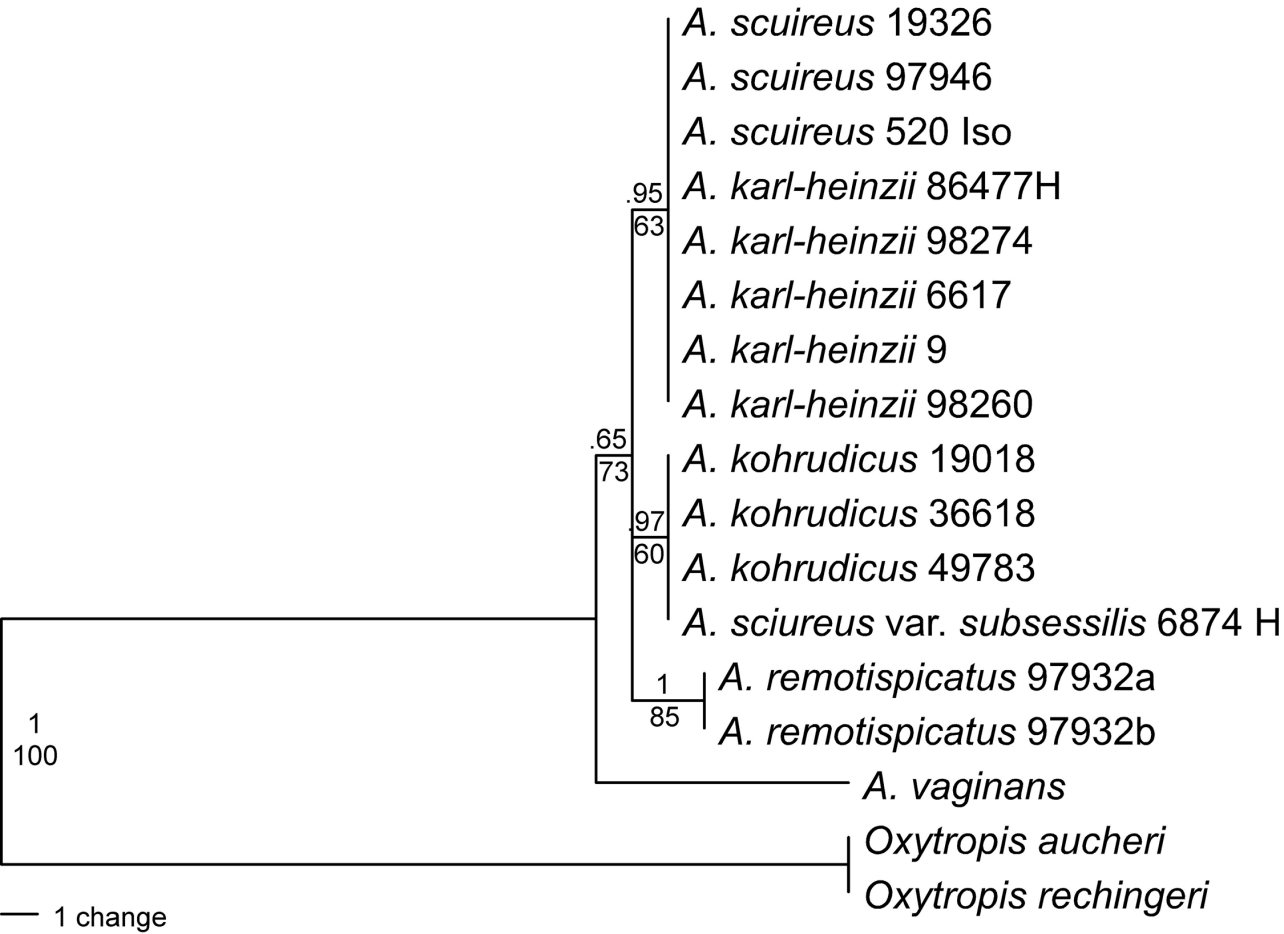
Single most parsimonious tree (tree length = 52 steps, consistency index = 1) derived from a phylogenetic analysis of sequence data of the nuclear ITS region. An identical tree topology was obtained with Bayesian phylogenetic inference. Numbers above branches indicate posterior probabilities of the Bayesian analysis, below the bootstrap values (%) for the parsimony analysis are given.

To infer the phylogenetic positions of *A*. *kohrudicus*/*A*. *sciureus* var. *subsessilis* and of the newly discovered populations from Zanjan, 17 ITS sequences were analyzed and resulted in an alignment length of 606 base pairs. In the parsimony analysis as well as in the Bayesian phylogenetic tree (Fig 4) the specimens of *A*. *kohrudicus* (19018, 36618, and 49783) group together with the holotype specimen of *A*. *sciureus* var. *subsessilis* (6874), while the specimens belonging to *A*. *sciureus* (19326, 97946 and isotype specimen 520) fall in a clade with *A*. *karl-heinzii*. The individuals of the newly discovered Zanjan species *A*. *remotispicatus* (97932a, b) formed a third clade. All three groups belong to a polytomy in the tree for which *A*. *vaginans* is the sister taxon.

## Discussion and Conclusion for New Species

**Fig 5:**
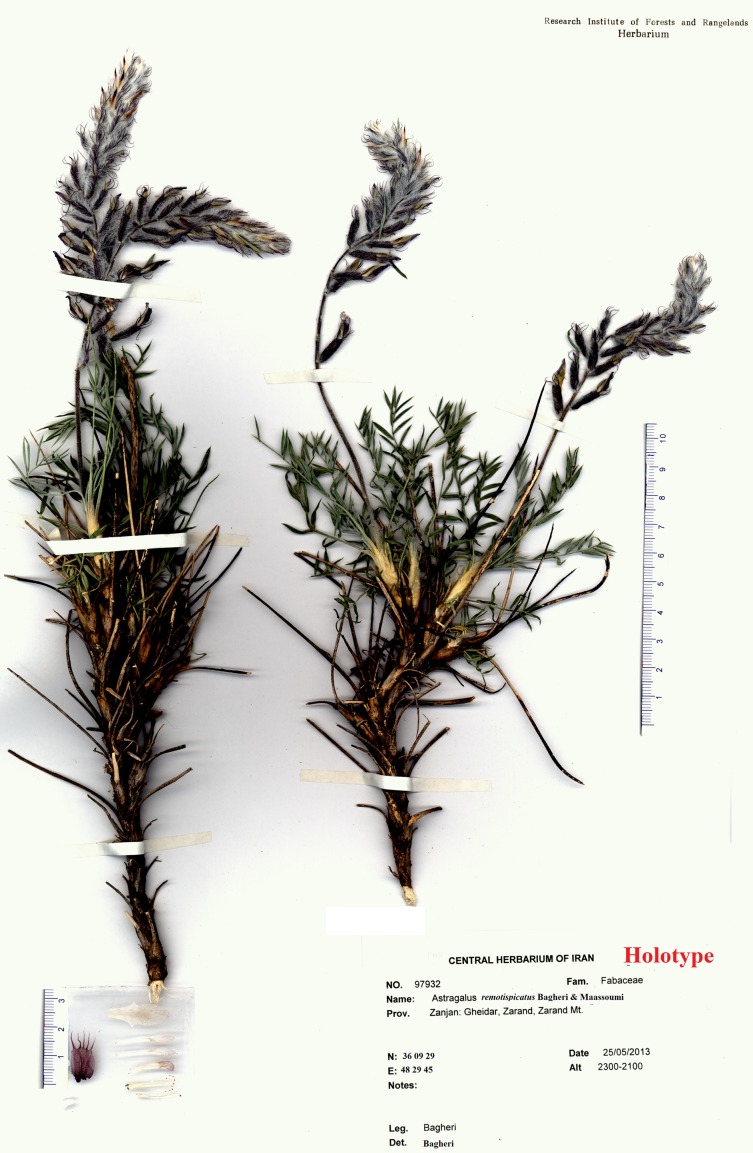
Holotype of *Astragalus remotispicatus* (Bagheri 97932).

**Fig 6:**
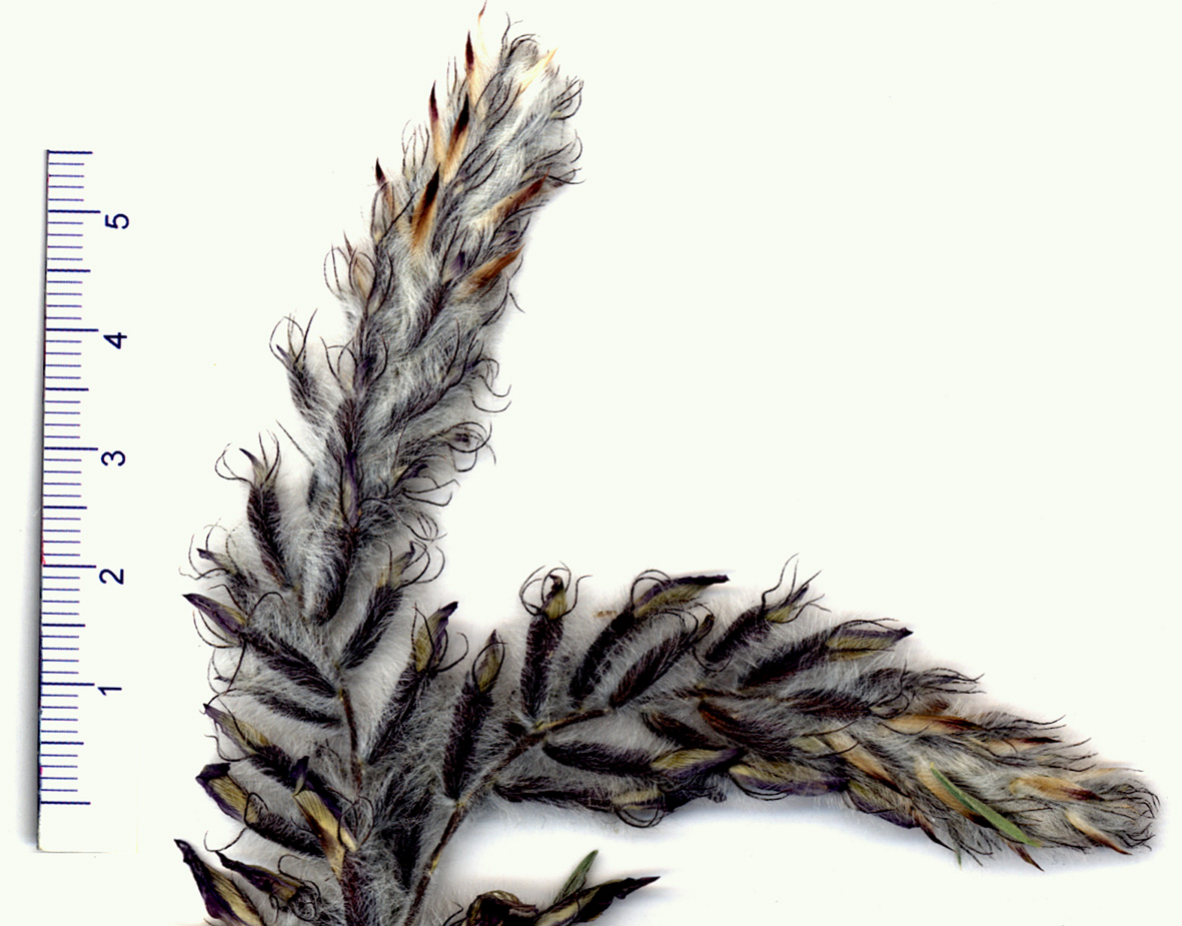
Close up of the inflorescence.

**Fig 7:**
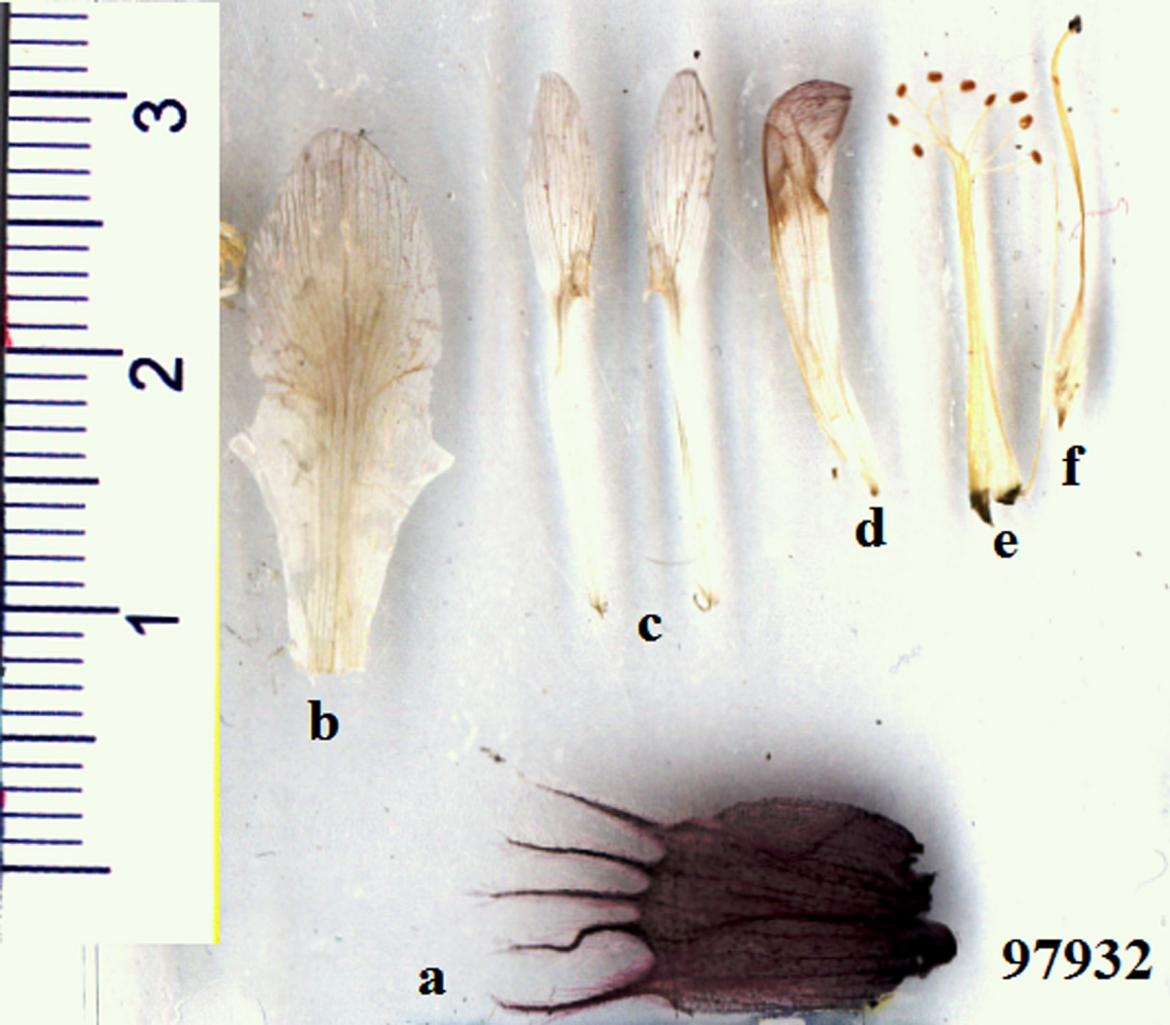
Segments of flowers including: a) Calyx, b) Standard, c) Wings, d) Keel, e) Stamens and f) Ovary.

***Astragalus remotispicatus*** Bagheri & Maassoumi *sp*.*nov*. [urn:lsid:ipni.org:names: 77152887–1] (Figs 5–7).

Type: IRAN. Zanjan province: Gheidar, Zarand village, Zarand Mt. 36° 09´ 29”N, 48° 29´ 45”E, 2100–2300 m, 25 May 2013, Bagheri 97932 (holotype TARI, isotypes HUI, TARI).

### Diagnosis

Differt ab *Astragalus karl-heinzii* stipulis 12–20 mm (nec 17 mm) longis, petiole ca. 12 adnatis (nec 7 mm) longis, pedunculis 5–7 (nec 2–2.5) cm longis, bracteis plerumque caducis, paucis persistentis in recemi juvenis, 8–9 × 3–4 (nec persistent, 14–15 × 4–6 mm) longis, calycis 13–16 mm (nec ca. 21 mm) longis, dense pilosis, usque ad 4 mm longis (nec 1.5–2.5 mm) longis. Ab *Astragalus rubrostriatus* stipulis 12–20 mm (nec 7–12 mm) longis, petiole ca. 12 mm adnatis (nec 4–7 mm) longis, pedunculis 5–7 (nec 3.5–15) cm longis, bracteis 8–9 × 3–4 (nec 8–20 × 3–8 mm) longis, calycis valde nigro violaceis (nec albido luteo, plerumque paraliter purpuro nervosis).

### Description

Subshrub, caespitose, 25–35 cm tall. Stems in the older parts ligneous, up to 5 cm long, branching from the base and covered with blackish bark and the remnants of the old petioles and stipules; younger stems in the current year ca. 2.5 cm long. Stipules membranous, hyaline at the apex, 12–20 mm long, triangular-acuminate, adnate to the petiole for up to 12 mm, very shortly connate, glabrous or ciliate at the margins. Leaves including petiole 3–7 cm; petiole up to 1.5–3 cm, both petiole and rachis densely covered with appressed white hairs 0.4–0.8 mm long. Leaflets opposite, in 6–8 pairs, the indumentum shining silvery and leaflet surface greenish; narrowly elliptic, 8–16 × 1–2.5 mm, acuminate, pungent, with a cusp 0.5–1 mm, on both sides densely covered with appressed white hairs 0.4–0.8 mm and with few spreading hairs up to 1 mm, partly complicate, terminal leaflets modified to a spine. Inflorescence a terminal raceme; peduncle 5–7 cm, at the apex part purplish, densely covered with erect, white hairs up to 2 mm and with very short spreading hairs up to 0.5 mm. Racemes cylindrical, 7–11 cm long, ca. 1.5–2.5 cm wide, loosely to remotely many-flowered, axis densely covered with erect to spreading hairs up to 2 mm long, the flowers very shortly pedicellate. Bracts mostly caducous, few persistent at the apex, elliptic, membranous, acuminate, 8–9 × 3–4 mm, glabrous, at the apex and central part violet, ciliate at the margins. Calyx inflated, 13–16 mm long, very dark violet, densely covered with erect to spreading white hairs up to 4 mm; teeth subulate, dark violet, 5–7 mm. Petals purple to violet, at the apex pale whitish, glabrous. Standard 17–22 mm long; blade 5–8 mm wide, slightly emarginate at the apex, obtusely hastate-auriculate at base, below the middle slightly constricted, gradually narrowed into the rather wide claw. Wing ca. 18–21 mm long; blade narrowly oblong to elliptic, obtuse, 7–9 × 1.5–3 mm; auricle ca. 0.8 mm; claw 9–13 mm. Keel 14–17 mm long; blade obliquely obovate, subacute, 4–6 × 2–3 mm; auricle indistinct; claw 9–10 mm. The claws of the wings and the keel are adnate to the staminal tube for 0.5–1 mm. Ovary 13–16 mm, sessile, hairy, stigma capitate. Stamens 14–17 mm, diadelphous, 9 + 1, the connate stamens free in the upper 3–4 mm. Fruit unknown.

### Etymology

The specific epithet “remotispicatus” refers to lax inflorescence of the new species.

### Taxonomic remarks

*Astragalus remotispicatus* is a rare endemic of northwestern Iran and known only from the type locality. It grows on rocky slopes. This new species has been found at elevations of 2100–2300 m. *Astragalus remotispicatus* belongs to *A*. sect. *Hymenostegis* and resembles *A*. *karl-heinzii* by having a lax inflorescence and purple calyx (Fig 5). However, it differs by some distinct features (see Table 4) from the latter. In addition, *A*. *remotispicatus* is different from *A*. *rubrostriatus* Bunge by its calyx color that is whitish to creamy (vs. very dark violet in the latter). Also *A*. *rubrostriatus* is endemic to northern and northwestern Iran.

**Table 4:** Differences between *Astragalus remotispicatus*, *A*. *karl-heinzii* and *A*. *rubrostriatus*.

Species / Characteristic	*A*. *remotispicatus*	*A*. *karl-heinzii*	*A*. *rubrostriatus*
**Stipules texture**	membranous	hyaline-membranous	hyaline-membranous
**Stipules length**	12–20 mm long	17 mm	7–12 mm
**Adnate to the petiole**	up to 12 mm	ca. 7 mm	4–7 mm
**Peduncles length**	5–7 cm, as long as the leaves	2−2.5 cm, distinctly shorter than the leaves	3.5–15 cm
**Peduncles hairs**	erect, white hairs up to 2 mm and with very short spreading hairs up to 0.5 mm	short and long spreading hairs 0.5−1 mm	appressed hairs 0.5–1 mm, with some appressed to ascending hairs 1.5–3 mm mixed in
**Inflorescence length**	7–11 cm long	9−12 cm long	5–15 cm long
**Bracts**	mostly caducous, (few persistent at the apex) membranous, 8–9 × 3–4 mm	persistent, chartaceous, 14−15 × 4−6 mm	caducous, chartaceous, 8–20 × 3–8 mm
**Calyx length**	13–16 mm long	ca. 21 mm long	11–16 mm long
**Calyx color**	very dark violet	violet, mostly with purple nerves	whitish to creamy, mostly with purple nerves
**Calyx hairs**	erect to spreading white hairs up to 4 mm	short and long, erect to spreading hairs 1.5−2.5 mm	short hairs up to 0.5(–1) mm and with erect to spreading hairs 3–4(–5) mm

### Phenology

Flowering May and early June; fruiting June to July

## Discussion and Conclusion for New Synonym

Based on the comparison of the relevant diagnoses, type specimens, morphological features and supporting data of ITS sequencing we concluded that *A*. *sciureus* var. *subsessilis* has to be treated as a synonym of *A*. *kohrudicus* as follows:

***Astragalus kohrudicus*** Bunge, Mémoires de l'Academie Imperiale des Sciences de Saint Petersbourg. Ser. 7. St. Pétersbourg. 11(16): 67 (1868); 15(1): 109 (1869).

*= Astragalus sciureus* var. *subsessilis* Bornm. *syn*. *nov*.

## Supporting Information

S1 Text ITS Sequences of examined taxa in this study. (DOCX)
