# Supplementary material for: Molecular Phylogeny and Morphological Analysis Support a New Species and New Synonymy in Iranian Astragalus (Leguminosae)
Source: PLoS One. 2016 Mar 9;11(3):e0149726. doi: 10.1371/journal.pone.0149726 (PMC4784885; doi:10.1371/journal.pone.0149726)
Supplement: S1 Text — (DOCX) [file pone.0149726.s001.docx]

**Supporting Information file 1.** Sequences of examined taxa in this study.

Oxytropis_aucheri_AB051908.1 TCGATGCCTTACATGCAGACCAACTCGTGAATCTGTTTGAATACAAGGGGATGGCTCGGGGTGTTT-GGCACCACGGCCTCCCTTTGGGTAGGAGGGG-CGCGCATTGCGTTCCCC-TCCTGCCCGAACACAAACCCCGGCGCTGAATGCGCCAAGGAACAAAAATTCGATCAGTGCGCCCCGTCGGCCCGGAGACGGTGCTCCGGCGGTGGTGCCTTGACACATGATACATAATGACTCTCGGCAACGGATATCTAGGCTCTTGCATCGATGAAGAACGTAGCGAAATGCGATACTTGGTGTGAATTGCAGAATCCCGTGAACCATCGAGTCTTTGAACGCAAGTTGCGCCCGAAGCCATTTGGTTGAGGGCACGTCTGCCTGGGCGTCACATATCGTTGCCCGATGCCTATTGCACTGTGATAGGAATTTCTAGGGCGAAAGATGGCTTCCCGTGAGCGTTGTTGCCTCGCGGTTGGTTGAAAATCGAGTCCTTGGTAGGGTGTGCCATGATAGATGGTGGTCGAGTTTGCACGAGACCGATCATGTGTGCGCTCCCCAAAATATTGACTCTTTGACCCCACACGCGTCTTTTGACGCTCATGA

Oxytropis_rechingeri_AB741305.1 TCGATGCCTTACATGCAGACCAACTCGTGAATCTGTTTGAATACAAGGGGATGGCTCGGGGTGTTT-GGCACCACGGCCTCCCTTTGGGTAGGAGGGG-CGCGCATTGCGTTCCCC-TCCTGCCCGAACACAAACCCCGGCGCTGAATGCGCCAAGGAACAAAAATTCGATCAGTGCGCCCCGTCGGCCCGGAGACGGTGCTCCGGCGGTGGTGCCTTGACACATGATACATAATGACTCTCGGCAACGGATATCTAGGCTCTTGCATCGATGAAGAACGTAGCGAAATGCGATACTTGGTGTGAATTGCAGAATCCCGTGAACCATCGAGTCTTTGAACGCAAGTTGCGCCCGAAGCCATTTGGTTGAGGGCACGTCTGCCTGGGCGTCACATATCGTTGCCCGATGCCTATTGCACTGTGATAGGAATTTCTAGGGCGAAAGATGGCTTCCCGTGAGCGTTGTTGCCTCGCGGTTGGTTGAAAATCGAGTCCTTGGTAGGGTGTGCCATGATAGATGGTGGTCGAGTTTGCACGAGACCGATCATGTGTGCGCTCCCCAAAATATTGACTCTTTGACCCCACACGCGTCTTTTGACGCTCATGA

A._kohrudicus_19018 TCGATGCCTTACATGCAGACCAACTCGTGAATTTGTTTGAATACATAGGGATGGCACGGG-TGTTTTGGCACCACGGCCTCCCTTTGGGTAGGAGGGGGAGCTCACTGCGTTCCCGCTCATGCCCGAACACAAACCCCGGCGTTCAATGCGCCAAGGAACTAAAATTTGATCAATGCGCCCCGTCGGCCCGGAGACGGTGCTTTGGCGGTGGTGCCTTGTCACATGATACAGAATGACTCTCGGCAACGGATATCTAGGCTCTTGCATCGATGAAGAACGTAGCGAAATGCGATACTTGGTGTGAATTGCAGAATCCCGTGAACCATCGAGTCTTTGAACGCAAGTTGCGCCCGAAGCCATCAGGTTGAGGGCACGTCTGCCTGGGCGTCACATATCGTTGCCCGATGCCTATTGCAGTGCAATAGGAATTTCTAGGGCGAATGATGGCTTCCCGTGAGCGTTGTTGCCTCGCGGTTGGTTGAAAATCGATTCCTTGGTAGGGTGTGCCATGATAGATGGTGGTCGAGTTAGGACGATACCGATCATGTGCATGCTCCCCAAGATATGGCCTCTATGACCC-ACACGTGTCTTTGGACGCTCATGA

A._kohrudicus_36618 TCGATGCCTTACATGCAGACCAACTCGTGAATTTGTTTGAATACATAGGGATGGCACGGG-TGTTTTGGCACCACGGCCTCCCTTTGGGTAGGAGGGGGAGCTCACTGCGTTCCCGCTCATGCCCGAACACAAACCCCGGCGTTCAATGCGCCAAGGAACTAAAATTTGATCAATGCGCCCCGTCGGCCCGGAGACGGTGCTTTGGCGGTGGTGCCTTGTCACATGATACAGAATGACTCTCGGCAACGGATATCTAGGCTCTTGCATCGATGAAGAACGTAGCGAAATGCGATACTTGGTGTGAATTGCAGAATCCCGTGAACCATCGAGTCTTTGAACGCAAGTTGCGCCCGAAGCCATCAGGTTGAGGGCACGTCTGCCTGGGCGTCACATATCGTTGCCCGATGCCTATTGCAGTGCAATAGGAATTTCTAGGGCGAATGATGGCTTCCCGTGAGCGTTGTTGCCTCGCGGTTGGTTGAAAATCgATTCCTTGGTAGGGTGTGCCATGATAGATGGTGGTCGAGTTAGGACGATACCGATCATGTGCATGCTCCCCAAGATATGGCCTCTATGACCC-ACACGTGTCTTTGGACGCTCATGA

A._kohrudicus_49783 TCGATGcCTTACATGCAGACCAACTCGTGAATTTGTTTGAATACATAGGGATGGCACGGG-TGTTTTGGCACCACGGCCTCCCTTTGGGTAGGAGGGGGAGCTCACTGCGTTCCCGCTCATGCCCGAACACAAACCCCGGCGTTCAATGCGCCAAGGAACTAAAATTTGATCAATGCGCCCCGTCGGCCCGGAGACGGTGCTTTGGCGGTGGTGCCTTGTCACATGATACAGAATGACTCTCGGCAACGGATATCTAGGCTCTtGCATCGATGAAGAACGTAGCGAAATGCGATACTTGGTGTGAATTGCAGAATCCCGTGAACCATCGAGTCTTTGAACGCAAGTTGCGCCCGAAGCCATCAGGTTGAGGGCACGTCTGCCTGGGCGTCACATATCGTTGCCCGATGCCTATTGCAGTGCAATAGGAATTTCTAGGGCGAATGATGGCTTCCCGTGAGCGTTGTTGCCTCGCGGTTGGTTGAAAATCGATTCCTTGGTAGGGTGTGCCATGATAGATGGTGGTCGAGTTAGGACGATACCGATCATGTGCATGCTCCCCAAGATATGGCCTCTATGACCC-ACACGTGTCTTTGGACGCTCATGA

A._sciureus_var_subsessilis_6874_H TCGATGCCTTACATGCAGACCAACTCGTGAATTTGTTTGAATACATAGGGATGGCACGGG-TGTTTTGGCACCACGGCCTCCCTTTGGGTAGGAGGGGGAGCTCACTGCGTTCCCGCTCATGCCCGAACACAAACCCCGGCGTTCAATGCGCCAAGGAACTAAAATTTGATCAATGCGCCCCGTCGGCCCGGAGACGGTGCTTTGGCGGTGGTGCCTTGTCACATGATACAGAATGACTCTCGGCAACGGATATCTAGGCTCTTGCATCGATGAAGAACGTAGCGAAATGCGATACTTGGTGTGAATTGCAGAATCCCGTGAACCATCGAGTCTTTGAACGCAAGTTGCGCCCGAAGCCATCAGGTTGAGGGCACGTCTGCCTGGGCGTCACATATCGTTGCCCGATGCCTATTGCAGTGCAATAGGAATTTCTAGGGCGAATGATGGCTTCCCGTGAGCGTTGTTGCCTCGCGGTTGGTTGAAAATCGATTCCTTGGTAGGGTGTGCCATGATAGATGGTGGTCGAGTTAGGACGATACCGATCATGTGCATGCTCCCCAAGATATGGCCTCTATGACCC-ACACGTGTCTTTGGACGCTCATGA

A._scuireus_19326 TCGATGCCTTACATGCAGACCAACTCGTGAATTTGTTTGAATACATAGGGATGGCACGGG-TGTTTTGGCACCACGGCCTCCCTTTGGGTAGGAGGGGGAGCTCACTGCGTTCCCGCTCATGCCCGAACACAAACCCCGGCGTTCAATGCGCCAAGGAACTAAAATTTGATCAATGCACCCCGTCGGCCCGGAGACGGTGCTTTGGCGGTGGTGCCTTGTCACATGATACAGAATGACTCTCGGCAACGGATATCTAGGCTCTTGCATCGATGAAGAACGTAGCGAAATGCGATACTTGGTGTGAATTGCAGAATCCCGTGAACCATCGAGTCTTTGAACGCAAGTTGCGCCCGAAGCCATCAGGTTGAGGGCACGTCTGCCTGGGCGTCACATATCGTTGCCCGATGCCTATTGCAGTGCAATAGGAATTTCTAGGGCGAATGATGGCTTCCCGTGAGCGTTGTTGCCTCGCGGTTGGTTGAAAATCGAGTCCTTGGTAGGGTGTGCCATGATAGATGGTGGTCGAGTTAGGACGATACCGATCATGTGCATGCTCCCCAAGATATGGCCTCTATGACCC-ACACGTGTCTTTGGACGCTCATGA

A._scuireus_97946 TCGATGCCTTACATGCAGACCAACTCGTGAATTTGTTTGAATACATAGGGATGGCACGGG-TGTTTTGGCACCACGGCCTCCCTTTGGGTAGGAGGGGGAGCTCACTGCGTTCCCGCTCATGCCCGAACACAAACCCCGGCGTTCAATGCGCCAAGGAACTAAAATTTGATCAATGCACCCCGTCGGCCCGGAGACGGTGCTTTGGCGGTGGTGCCTTGTCACATGATACAGAATGACTCTCGGCAACGGATATCTAGGCTCTTGCATCGATGAAGAACGTAGCGAAATGCGATACTTGGTGTGAATTGCAGAATCCCGTGAACCATCGAGTCTTTGAACGCAAGTTGCGCCCGAAGCCATCAGGTTGAGGGCACGTCTGCCTGGGCGTCACATATCGTTGCCCGATGCCTATTGCAGTGCAATAGGAATTTCTAGGGCGAATGATGGCTTCCCGTGAGCGTTGTTGCCTCGCGGTTGGTTGAAAATCGAGTCCTTGGTAGGGTGTGCCATGATAGATGGTGGTCGAGTTAGGACGATACCGATCATGTGCATGCTCCCCAAGATATGGCCTCTATGACCC-ACACGTGTCTTTGGACGCTCATGA

A._scuireus_520_Iso TCGATGCCTTACATGCAGACCAACTCGTGAATTTGTTTGAATACATAGGGATGGCACGGG-TGTTTTGGCACCACGGCCTCCCTTTGGGTAGGAGGGGGAGCTCACTGCGTTCCCGCTCATGCCCGAACACAAACCCCGGCGTTCAATGCGCCAAGGAACTAAAATTTGATCAATGCACCCCGTCGGCCCGGAGACGGTGCTTTGGCGGTGGTGCCTTGTCACATGATACAGAATGACTCTCGGCAACGGATATCTAGGCTCTTGCATCGATGAAGAACGTAGCGAAATGCGATACTTGGTGTGAATTGCAGAATCCCGTGAACCATCGAGTCTTTGAACGCAAGTTGCGCCCGAAGCCATCAGGTTGAGGGCACGTCTGCCTGGGCGTCACATATCGTTGCCCGATGCCTATTGCAGTGCAATAGGAATTTCTAGGGCGAATGATGGCTTCCCGTGAGCGTTGTTGCCTCGCGGTTGGTTGAAAATCGAGTCCTTGGTAGGGTGTGCCATGATAGATGGTGGTCGAGTTAGGACGATACCGATCATGTGCATGCTCCCCAAGATATGGCCTCTATGACCC-ACACGTGTCTTTGGACGCTCATGA

A._karl_heinzii_86477H TCGATGCCTTACATGCAGACCAACTCGTGAATTTGTTTGAATACATAGGGATGGCACGGG-TGTTTTGGCACCACGGCCTCCCTTTGGGTAGGAGGGGGAGCTCACTGCGTTCCCGCTCATGCCCGAACACAAACCCCGGCGTTCAATGCGCCAAGGAACTAAAATTTGATCAATGCACCCCGTCGGCCCGGAGACGGTGCTTTGGCGGTGGTGCCTTGTCACATGATACAGAATGACTCTCGGCAACGGATATCTAGGCTCTTGCATCGATGAAGAACGTAGCGAAATGCGATACTTGGTGTGAATTGCAGAATCCCGTGAACCATCGAGTCTTTGAACGCAAGTTGCGCCCGAAGCCATCAGGTTGAGGGCACGTCTGCCTGGGCGTCACATATCGTTGCCCGATGCCTATTGCAGTGCAATAGGAATTTCTAGGGCGAATGATGGCTTCCCGTGAGCGTTGTTGCCTCGCGGTTGGTTGAAAATCGAGTCCTTGGTAGGGTGTGCCATGATAGATGGTGGTCGAGTTAGGACGATACCGATCATGTGCATGCTCCCCAAGATATGGCCTCTATGACCC-ACACGTGTCTTTGGACGCTCATGA

A._karl_heinzii_98274 TCGATGCCTTACATGCAGACCAACTCGTGAATTTGTTTGAATACATAGGGATGGCACGGG-TGTTTTGGCACCACGGCCTCCCTTTGGGTAGGAGGGGGAGCTCACTGCGTTCCCGCTCATGCCCGAACACAAACCCCGGCGTTCAATGCGCCAAGGAACTAAAATTTGATCAATGCACCCCGTCGGCCCGGAGACGGTGCTTTGGCGGTGGTGCCTTGTCACATGATACAGAATGACTCTCGGCAACGGATATCTAGGCTCTTGCATCGATGAAGAACGTAGCGAAATGCGATACTTGGTGTGAATTGCAGAATCCCGTGAACCATCGAGTCTTTGAACGCAAGTTGCGCCCGAAGCCATCAGGTTGAGGGCACGTCTGCCTGGGCGTCACATATCGTTGCCCGATGCCTATTGCAGTGCAATAGGAATTTCTAGGGCGAATGATGGCTTCCCGTGAGCGTTGTTGCCTCGCGGTTGGTTGAAAATCGAGTCCTTGGTAGGGTGTGCCATGATAGATGGTGGTCGAGTTAGGACGATACCGATCATGTGCATGCTCCCCAAGATATGGCCTCTATGACCC-ACACGTGTCTTTGGACGCTCATGA

A._karl_heinzii_6617 TCGATGCCTTACATGCAGACCAACTCGTGAATTTGTTTGAATACATAGGGATGGCACGGG-TGTTTTGGCACCACGGCCTCCCTTTGGGTAGGAGGGGGAGCTCACTGCGTTCCCGCTCATGCCCGAACACAAACCCCGGCGTTCAATGCGCCAAGGAACTAAAATTTGATCAATGCACCCCGTCGGCCCGGAGACGGTGCTTTGGCGGTGGTGCCTTGTCACATGATACAGAATGACTCTCGGCAACGGATATCTAGGCTCTTGCATCGATGAAGAACGTAGCGAAATGCGATACTTGGTGTGAATTGCAGAATCCCGTGAACCATCGAGTCTTTGAACGCAAGTTGCGCCCGAAGCCATCAGGTTGAGGGCACGTCTGCCTGGGCGTCACATATCGTTGCCCGATGCCTATTGCAGTGCAATAGGAATTTCTAGGGCGAATGATGGCTTCCCGTGAGCGTTGTTGCCTCGCGGTTGGTTGAAAATCGAGTCCTTGGTAGGGTGTGCCATGATAGATGGTGGTCGAGTTAGGACGATACCGATCATGTGCATGCTCCCCAAGATATGGCCTCTATGACCC-ACACGTGTCTTTGGACGCTCATGA

A._karl_heinzii_9 TCGATGCCTTACATGCAGACCAACTCGTGAATTTGTTTGAATACATAGGGATGGCACGGG-TGTTTTGGCACCACGGCCTCCCTTTGGGTAGGAGGGGGAGCTCACTGCGTTCCCGCTCATGCCCGAACACAAACCCCGGCGTTCAATGCGCCAAGGAACTAAAATTTGATCAATGCACCCCGTCGGCCCGGAGACGGTGCTTTGGCGGTGGTGCCTTGTCACATGATACAGAATGACTCTCGGCAACGGATATCTAGGCTCTTGCATCGATGAAGAACGTAGCGAAATGCGATACTTGGTGTGAATTGCAGAATCCCGTGAACCATCGAGTCTTTGAACGCAAGTTGCGCCCGAAGCCATCAGGTTGAGGGCACGTCTGCCTGGGCGTCACATATCGTTGCCCGATGCCTATTGCAGTGCAATAGGAATTTCTAGGGCGAATGATGGCTTCCCGTGAGCGTTGTTGCCTCGCGGTTGGTTGAAAATCGAGTCCTTGGTAGGGTGTGCCATGATAGATGGTGGTCGAGTTAGGACGATACCGATCATGTGCATGCTCCCCAAGATATGGCCTCTATGACCC-ACACGTGTCTTTGGACGCTCATGA

A._karl_heinzii_98260 TCGATGCCTTACATGCAGACCAACTCGTGAATTTGTTTGAATACATAGGGATGGCACGGG-TGTTTTGGCACCACGGCCTCCCTTTGGGTAGGAGGGGGAGCTCACTGCGTTCCCGCTCATGCCCGAACACAAACCCCGGCGTTCAATGCGCCAAGGAACTAAAATTTGATCAATGCACCCCGTCGGCCCGGAGACGGTGCTTTGGCGGTGGTGCCTTGTCACATGATACAGAATGACTCTCGGCAACGGATATCTAGGCTCTTGCATCGATGAAGAACGTAGCGAAATGCGATACTTGGTGTGAATTGCAGAATCCCGTGAACCATCGAGTCTTTGAACGCAAGTTGCGCCCGAAGCCATCAGGTTGAGGGCACGTCTGCCTGGGCGTCACATATCGTTGCCCGATGCCTATTGCAGTGCAATAGGAATTTCTAGGGCGAATGATGGCTTCCCGTGAGCGTTGTTGCCTCGCGGTTGGTTGAAAATCGAGTCCTTGGTAGGGTGTGCCATGATAGATGGTGGTCGAGTTAGGACGATACCGATCATGTGCATGCTCCCCAAGATATGGCCTCTATGACCC-ACACGTGTCTTTGGACGCTCATGA

A._remotispicatus_97932a TCGATGCCTTACATGCAGACCAACTCGTGAATTTGTTTGAATACATAGGGATGGCACGGG-TGTTTTGGCACCACGGCCTCCCTTTGGGTAGGAGGGGGAGCTCACTGCGTTCCCGCTCATGCCCGAACACAAACCCCGGCGTTCAATGCGCCAAGGAACTAAAATTTGATCAATGCGCCCCGTCGGCCCGGAGACGGTGCTTTGGCGGTGGTGCCTTGTCACATGATACAGAATGACTCTCGGCAACGGATATCTAGGCTCTTGCATCGATGAAGAACGTAGCGAAATGCGATACTTGGTGTGAATTGCAGAATCCCGTGAACCATCGAGTCTTTGAACGCAAGTTGCGCCCGAAGCCATCAGGTTGAGGGCACGTCTGCCTGGGCGTCACATATCGTTGCCCGATGCCTATTGCAGTGCAATAGGAATTTCTAGGGCGAATGATGGCTTCCCGTGAGCGTTGTTGCCTCGCGGTTGGTTGAAAATCGAGTCCTTGGTAGGGTGTGCCATGATAGATGGTGGTCGAGTTAGGACGATACCGATCATGTGCTTGCCCCCCAAGATATGGCCTCTATGACCC-ACACGTGTCTTTGGACGCTCATGA

A._remotispicatus_97932b TCGATGCCTTACATGCAGACCAACTCGTGAATTTGTTTGAATACATAGGGATGGCACGGG-TGTTTTGGCACCACGGCCTCCCTTTGGGTAGGAGGGGGAGCTCACTGCGTTCCCGCTCATGCCCGAACACAAACCCCGGCGTTCAATGCGCCAAGGAACTAAAATTTGATCAATGCGCCCCGTCGGCCCGGAGACGGTGCTTTGGCGGTGGTGCCTTGTCACATGATACAGAATGACTCTCGGCAACGGATATCTAGGCTCTTGCATCGATGAAGAACGTAGCGAAATGCGATACTTGGTGTGAATTGCAGAATCCCGTGAACCATCGAGTCTTTGAACGCAAGTTGCGCCCGAAGCCATCAGGTTGAGGGCACGTCTGCCTGGGCGTCACATATCGTTGCCCGATGCCTATTGCAGTGCAATAGGAATTTCTAGGGCGAATGATGGCTTCCCGTGAGCGTTGTTGCCTCGCGGTTGGTTGAAAATCGAGTCCTTGGTAGGGTGTGCCATGATAGATGGTGGTCGAGTTAGGACGATACCGATCATGTGCTTGCCCCCCAAGATATGGCCTCTATGACCC-ACACGTGTCTTTGGACGCTCATGA

A._vaginans_AB908466.1 TCGATGCCTTACATGCAGACCAACTCGTGAATTTGTTTGAATACATAGGGATGGCACGGG-TGTTTTGGCACCACGGCCTCTCTTTGGGTAGGAGGGGGAGCTCACTGCGTTCCCGCTCATGCCCGAACACAAACCCCGGCGTTCAATGCGCCAAGGAACTATAATTCGATCAATGCGCCCCGTCGGCCCGGAGACGGTGCTTTGGCGGCGGTGCCTTGTCACATGATACAGAATGACTCTCGGCAACGGATATCTAGGCTCTTGCATCGATGAAGAACGTAGCGAAATGCGATACTTGGTGTGAATTGCAGAATCCCGTGAACCATCGAGTCTTTGAACGCAAGTTGCGCCCGAAGCCATCAGGTTGAGGGCACGTCTGCCTGGGCGTCACATATCGTTGCCCGATGCCTATTGCAGTGCAATAGGAATTTTTAGGGCGAATGATGGCTTCCCGTGAGCGTTGTTGCCTCGCGGTTGGTTGAAAATCGAGTCCTTGGTAGGATGTGCCATGATAGATGGTGGTCGAGTTAGGACGATACCGATCATGTGCATTCTCCCCAAGATATGGCCTCTGTGACCC-ACACGTGTCTTTGGACGCTCATGA
